# Supplementary material for: A high-throughput cytotoxicity screening platform reveals agr-independent mutations in bacteraemia-associated Staphylococcus aureus that promote intracellular persistence
Source: eLife. 2023 Jun 8;12:e84778. doi: 10.7554/eLife.84778 (PMC10259494; doi:10.7554/eLife.84778)
Supplement: Supplementary file 7. [file elife-84778-supp7.docx]

| **Name** | **Oligonucleotide sequence (5' to 3')** | **Amino acid change** |
| --- | --- | --- |
| *mfd*-W568*-F2 | GGCAGTGAATGAAAAAAAACAAAAGC | ***mfd*-W568*** |
| *mfd*-W568*-R2 | GCTTTTGTTTTTTTTCATTCACTGCC |  |
| pIMAY-Z-*mfd*-W568*-F | CCTCACTAAAGGGAACAAAAGCTGGGTACCGATTGAAGGCTATCCTGTCAC |  |
| pIMAY-Z-*mfd-*W568*-R | CGACTCACTATAGGGCGAATTGGAGCTCCTAGGTATTGGGGTTGCAGTC |  |
| *ausA*-K2308FS-F2 | GCCGGTGAGAATGTGTTAAGTGC | ***ausA*-K2308FS** |
| *ausA*-K2308FS-R2 | GCACTTAACACATTCTCACCGGC |  |
| pIMAY-Z-*ausA*-K2308FS-F | CCTCACTAAAGGGAACAAAAGCTGGGTACCGTTGGTGATTTCGAGTGTATGG |  |
| pIMAY-Z-*ausA*-K2308FS-R | CGACTCACTATAGGGCGAATTGGAGCTCGTACGCCAGTCTAAACGTTGTG |  |
| *alr*-K354FS-F2 | CATGATTCTTTAAAAAGGAGTACATTTC | ***alr*-K354FS** |
| *alr*-K354FS-R2 | GAAATGTACTCCTTTTTAAAGAATCATG |  |
| pIMAY-Z-*alr*-K354FS-F | CCTCACTAAAGGGAACAAAAGCTGGGTACCGCTGATGAATTCGATGTGTCAG |  |
| pIMAY-Z-*alr*-K354FS-R | CGACTCACTATAGGGCGAATTGGAGCTCCTGTACCTTCAATCTGTGAACC |  |
| *cydA*-R390C-F2 | GGATTGTTTGTGGTTATATGCG | ***cydA*-R390C** **(reversion)** |
| *cydA*-R390C-R2 | CGCATATAACCACAAACAATCC |  |
| pIMAY-Z-*cydA*-R390C-F | CCTCACTAAAGGGAACAAAAGCTGGGTACCCAGCCGGTATGACGATGG |  |
| pIMAY-Z-*cydA*-R390C-R | CGACTCACTATAGGGCGAATTGGAGCTCCTGCACCCAATCTAGATCG |  |
| *agrA*-E7K-R2 | TGGATCGTCTTTGCAAATGA | ***agrA*-E7K** |
| *agrA*-E7K-F2 | TCATTTGCAAAGACGATCCA |  |
| pIMAY-Z-*agrA*-E7K-R | CGACTCACTATAGGGCGAATTGGAGCTCGTTTCTCACCGATGCATAGC |  |
| *agrC*-G310FS-F2 | GGTGAAGTCGTGGTTTAGG | ***agrC*-G310FS** |
| *agrC*-G310FS-R2 | CCTAAACCACGACTTCACC |  |
| pIMAY-Z-*agrC*-G310FS-R | CGACTCACTATAGGGCGAATTGGAGCTCGGGCAATGAGTCTGTGAG |  |
| pIMAY-Z-*agrC*-G310FS-*agrA*-E7K-F | CCTCACTAAAGGGAACAAAAGCTGGGTACCGTTATTTCGACTATCTTACTGCTTAC | ***agrA-*E7K and *agrC*-G310FS** |

Primers used to introduce convergent mutations in BPH3370 by site-directed mutagenesis
